# Down regulation of lincRNA-p21 contributes to gastric cancer development through Hippo-independent activation of YAP

**DOI:** 10.18632/oncotarget.19130

**Published:** 2017-07-10

**Authors:** Ying Chen, Guoqing Wei, Hongwei Xia, Huangfei Yu, Qiuling Tang, Feng Bi

**Affiliations:** ^1^ Department of Medical Oncology, West China Hospital, Sichuan University, Chengdu, P.R. China; ^2^ State Key Laboratory of Biotherapy and Cancer Center, West China Hospital, Sichuan University, and Collaborative Innovation Center for Biotherapy, Chengdu, P.R. China

**Keywords:** long non-coding RNA, lincRNA-p21, gastric cancer, YAP, hippo

## Abstract

Long intergenic non-coding RNA p21 (lincRNA-p21), known as the direct transcriptional target of p53, was found down-regulated in several human solid tumors. However, little is known about the role of lincRNA-p21 in gastric cancer. The expression levels of lincRNA-p21 in tissue samples and cell lines were detected by qRT-PCR. MGC-803 and MKN-45 cells were transfected with siRNAs targeting lincRNA-p21 or control siRNAs to determine the effect of reduced lincRNA-p21 expression on tumorigenesis. We also overexpressed lincRNA-p21 in MGC-803 cells. Cell proliferation was measured by CCK-8 and Ethynyl-2-deoxyuridine (EdU) incorporation assays. Migration and invasion abilities of cells were measured by wound healing and transwell assay. We demonstrated that lincRNA-p21 was significantly reduced in gastric cancer tissues (p<0.001) compared with that in normal tissues and this lower level of lincRNA-p21 was significantly correlated with higher invasion depth grade (p=0.024), more distant metastasis (p=0.009) and advanced TNM stage (p=0.011). Further study revealed that knock down of lincRNA-p21 could promote malignant behavior of gastric cancer cells and induce epithelial to mesenchymal transition (EMT). Overexpressing lincRNA-p21 showed opposite effects. Moreover, knocking down lincRNA-p21 could elevate the expression of Yes associated protein (YAP), the core effector of Hippo signaling, by elevating mRNA levels and increasing its nucleus translocation instead of the canonical Hippo pathway. Overexpression experiments verified the regulation role of lincRNA-p21 in YAP expression. Collectively, these data suggest that lincRNA-p21 could serve as a potential biomarker and a vital therapeutic target in gastric cancer.

## INTRODUCTION

Gastric cancer (GC) is the most common gastrointestinal cancer in East Asia and ranks as the third leading type of cancer-related death worldwide [1, 2]. The mortality rate has remained at a rather high level during the latest 10 years in China [3, 4]. Therefore it is urgent that we identify novel biomarkers and their underlying molecular mechanisms in gastric carcinogenesis. These biomarkers may not only improve the early diagnosis and prognosis, but also may serve as molecular targets for gastric cancer therapy.

Long noncoding RNA (lncRNA), which is defined as transcripts composed of more than 200 nucleotides in length, have attracted broad attention recently [5–7]. Long intergenic non-coding RNA p21 (lincRNA-p21) is known as a direct transcriptional target of p53 and is ∼3kb in length. It mainly regulates the cell cycle and apoptosis [8]. Several studies have shown that lower expression levels of lincRNA-p21 are correlated with the poor prognosis of colorectal cancer and hepatocellular carcinoma [9, 10]. However, the role of lincRNA-p21 in gastric cancer remains largely unknown.

The Hippo pathway governs the organ size and cell growth rate [11]. Yes-associated protein (YAP), the core effector of Hippo pathway, has long been considered an important proto-oncogene. The overexpression of YAP was found in multiple types of malignancies, including liver cancer, esophageal squamous cell carcinoma, non-small lung cancer and ovarian cancer [12–15, 18]. Although the regulation of YAP by the Hippo pathway has been heavily researched, how YAP is regulated by Hippo-independent pathways remains elusive.

Our study first confirmed the expression pattern of the lincRNA-p21 expression in GC tissues and cell lines. We then investigated the relationship between lincRNA-p21 levels and the malignant behavior of gastric cancer in two GC cell lines. The potential lincRNA-p21-dependent mechanisms correlated with EMT and YAP expression were further explored using *in vitro* experiments.

## RESULTS

### LincRNA-p21 is significantly down-regulated in gastric cancer

**Figure 1:**
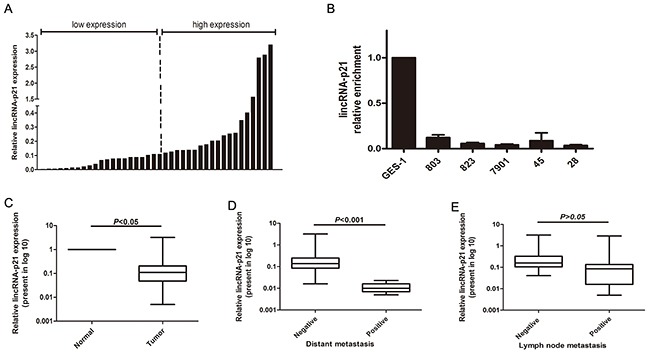
The expression level of lincRNA-p21 in GC tissues and GC cell lines The relative lincRNA-p21 expression level was measured by qRT-PCR **(A)** The data was shown as the fold change in the GC tissues compared with the paired normal tissues. Two groups were set according to the median ratio. **(B)** The lincRNA-p21 expression in GC cell and GES-1. **(C)** The statistical analysis of the lincRNA-p21 expression in GC tissues compared to the paired normal tissues. **(D)** The relationship betweenlincRNA-p21 expression and distant metastasis. **(E)** The relationship between lincRNA-p21 expression andlymph node metastasis. Bars represents for Standard Deviation (SD). All data showed a representative of three independent experiments.

We first measured the expression level of lincRNA-p21 by qRT-PCR in 40 pairs of gastric cancer tissues and adjacent non-cancerous tissues. We first expressed this paired group data as fold changes in the GC tissues relative to the paired normal tissues and then separated the paired tissues into low (n=21, ratio≤0.11) and high (n=19, ratio>0.11) expression groups according to the median ratio (Figure 1A). Our statistical analysis indicated that lincRNA-p21 was significantly down-regulated in tumor tissue samples compared to paired normal tissue (*p*<0.05) (Figure 1C). Further analysis indicated a significantly lower level of lincRNA-p21 in GC tissues with distant metastasis (*p*<0.001) (Figure 1D). However, the reduced lincRNA-p21 expression was not significantly correlated with lymph node metastasis (*p*>0.05) (Figure 1E). The comparison of the clinicopathological characteristics between relative low expression group and high expression group demonstrated that the lower expression level of lincRNA-p21 was correlated with higher invasion depth grade (*p*=0.024), distant metastasis (*p*=0.009) and more advanced TNM stage (*p*=0.011) (Table 1). Next, we performed qRT-PCR to examine the expression of lincRNA-p21 in gastric cancer cell lines. The results showed that lincRNA-p21 was significantly decreased in multiple gastric cancer cell lines compared with the normal gastric mucosal cell line GES-1 (Figure 1B).

**Table 1:** Correlation between lincRNA-p21 expression and clinicopathological features in gastric cancer patients

Clinical characteristics	Total number	LincRNA-p21 expression		*P* value
		low	high	
**Age**				
>60	18	7	11	0.107
<60	22	14	8	
**Gender**				
Male	25	13	12	0.597
Female	15	8	7	
**Invasion depth**				
T1	4	1	3	0.024*
T2	11	3	8	
T3	12	6	6	
T4	13	11	2	
**Lymphnode metastasis**				
0	16	5	11	0.057
1	13	7	6	
2	7	5	2	
3	4	4	0	
**Metastasis**				
Yes	7	7	0	0.009*
No	33	14	19	
**TNM stage**				
I	13	3	10	0.011*
II	12	6	6	
III	8	5	3	
IV	7	7	0	

* represents for p<0.05

### The effect of lincRNA-p21 on GC cell proliferation

**Figure 2:**
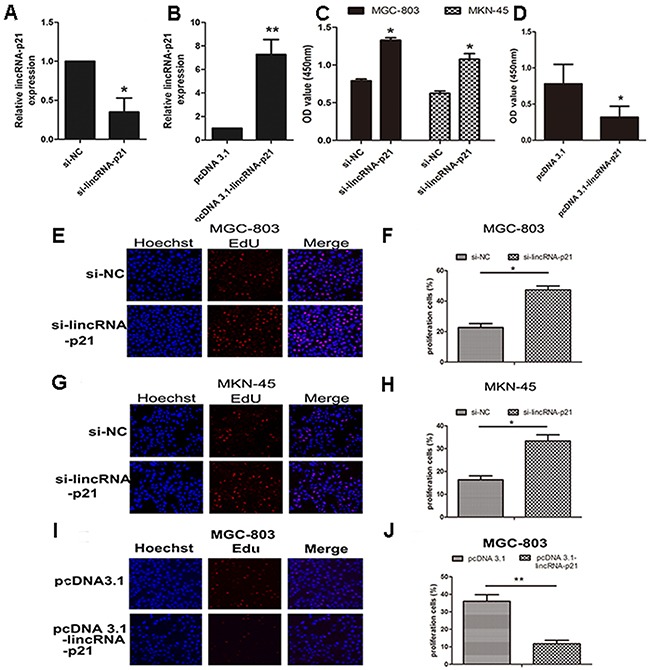
The effect of lincRNA-p21 on GC cell proliferation The knockdown efficiency **(A)** and overexpression **(B)** efficiency of lincRNA-p21 tested in MGC-803. CCK-8 kit was used to test the cell viability at 48h post-transfection of siRNA in both MGC-803 and MKN-45 **(C)**. CCK-8 data of pcDNA 3.1-lincRNA-p21 transfection in MGC-803 cell line **(D)**. Edu incorporation assay was performed to further test the cell proliferation at 48h post-transfection. Pictures of knockdown experiments in MGC-803 **(E)** and MKN-45 **(G)**. Pictures of overexpression experiments in MGC-803 cells **(I)**. Red labeled cells indicated as the proliferated cells. The percentage of the proliferated cells was also counted in both knockdown experiments **(F)** and **(H)** and overexpression experiments **(J)**. Bars represents for SD. All data shows a representative of three independent experiments. * represents for *p*<0.05, ** represents for *p*<0.01.

In order to study the biological function of lincRNA-p21 in gastric cancers, we performed knockdown and overexpression experiments. The expression level of lincRNA-p21 was significantly down-regulated in the si-lincRNA-p21-transfected cells (*p*<0.05, Figure 2A). Meanwhile, the expression level of lincRNA-p21 was significantly up-regulated in the cells transfected with a pcDNA 3.1-lincRNA-p21 overexpresssion vector (*p*<0.01, Figure 2B). CCK-8 assays demonstrated that the knockdown of lincRNA-p21 promoted cell growth in both MGC-803 and MKN-45 cells (Figure 2C) while lincRNA-p21 overexpression showed the opposite result (Figure 2D). Additionally, the Edu assay also showed enhanced cell proliferation ability in the MGC-803 (Figure 2E and 2F) and MKN-45 cells (Figure 2G and 2H) transfected with si-lincRNA-p21. In contrast, the cell proliferation ability of MGC-803 was impaired during lincRNA-p21 overexpression (Figure 2I and 2J).

### The relationship between lincRNA-p21 and EMT process

**Figure 3:**
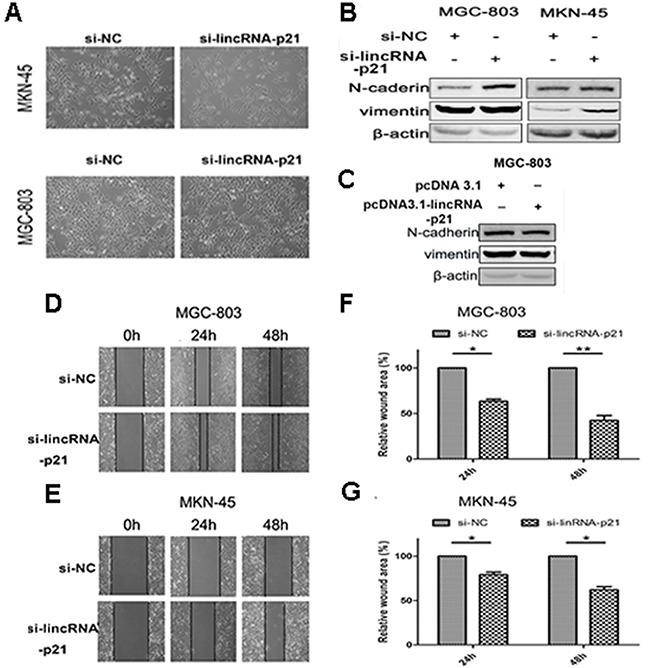
The effect of lincRNA-p21 on cell migration ability and EMT process The morphology change was observed at 48h post-transfection of siRNA in MGC-803 and MKN-45 cell lines by bright field microscopy (20×) **(A)**. The western blot result in knockdown experiment **(B)** and overexpression experiment **(C)**. Pictures of wound healing assay in knockdown experiments **(D)** and **(E)**. Relative wound area was calculated as (wound area at 24 or 48h/wound area at 0h), which was then normalized to si-NC group in MGC-803 **(F)** and MKN-45 **(G)**. Bars represents for SD. All data shows a representative of three independent experiments. * represents for p<0.05, ** represents for p<0.01.

**Figure 4:**
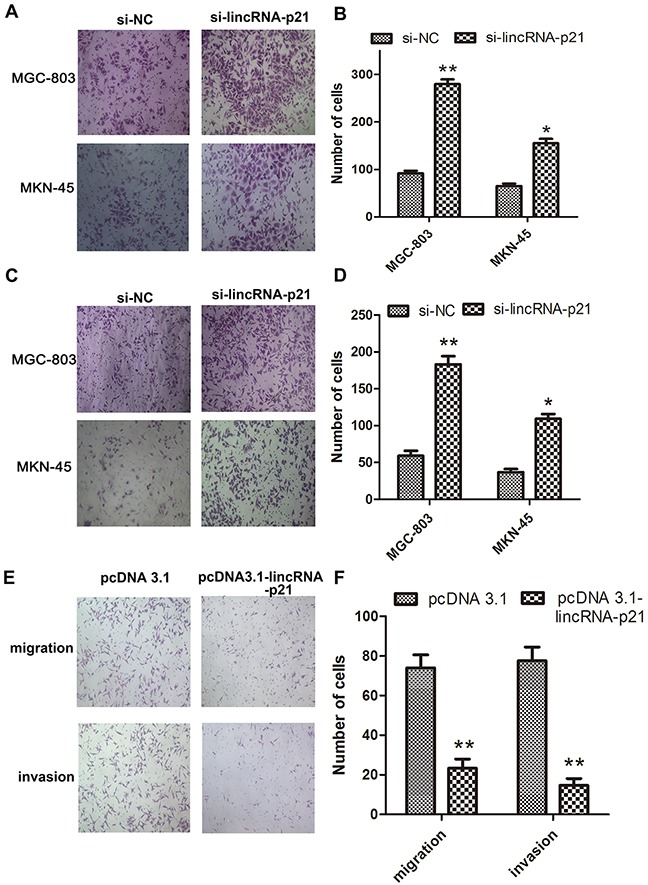
The effect of lincRNA-p21 on cell migration and invasion ability Representative pictures of transwell assays showed the change of thecell migration **(A)** and invasion **(C)** ability in knockdown experiments and in overexpression experiments **(E)**. Quantification data in knockdown experiments **(B)** and **(D)** as well as overexpression experiments were showed **(F)**. Bars represents for SD. All data shows a representative of three independent experiments. * represents for p<0.05, ** represents for p<0.01.

We next evaluated the effect of down-regulated lincRNA-p21 on the EMT process. We found that the morphology of both MGC-803 and MKN-45 dramatically changed at 48h post-transfection of si-lincRNA-p21. They lost their cell to cell contacts and acquired spindle-like appearance (Figure 3A). Our western blots showed that vimentin and N-cadherin were significantly elevated in si-lincRNA-p21 group (Figure 3B) while lincRNA-p21 overexpression showed an opposite result (Figure 3C). In wound healing assays, we showed that the wound area became narrower in si-lincRNA-p21 groups compared with control groups in both MGC-803 (Figure 3D) and MKN-45 (Figure 3E) cells. Quantitative analysis revealed that the decrease of the wound area was statistically significant at both 24h and 48h post-transfection time point in MGC-803 (Figure 3F) and MKN-45 (Figure 3G) cells. Transwell assays were also performed to test the cell migration and invasion ability. We observed that the knockdown of lincRNA-p21 lead to more cells passing through the chambers with or without the coated matrigel in MGC-803 and MKN-45 cells (Figure 4A and 4C). Further quantitative analysis showed a significantly increased number of cells passing through the chamber in MGC-803 and MKN-45 experiments during knockdown of lincRNA-p21 (Figure 4B and 4D). In lincRNA-p21 overexpression experiments, fewer cells passed through the chambers compare to control groups (Figure 4E) and quantitative analysis supported this finding (Figure 4F).

### The role of lincRNA-p21 in the regulation of YAP expression

**Figure 5:**
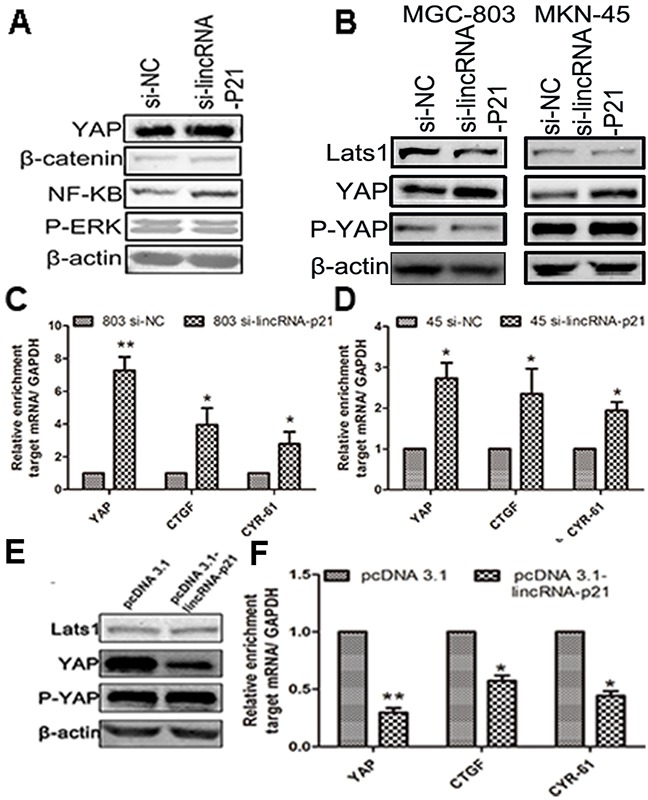
The regulation of lincRNA-p21 on YAP expression was performed in a Hippo independent manner **(A)** The western blot showed elevated expression of β-catenin, YAP and NF-κB in si-lincRNA-p21 group. **(B)** The western blot result indicated that the YAP and P-YAP were up-regulated in si-lincRNA-p21 group while the Lats1 remained almost unchangeable. Knocking down of lincRNA-p21 resulted in the elevation of mRNA levels of YAP, CTGF and CYR-61 in **(C)** and MKN-45 **(D)** cells. **(E)** The western blot result showed that the YAP and P-YAP were down-regulated in MGC-803 cells transfected with pcDNA 3.1-lincRNA-p21 while the Lats1 still remains unchangeable. **(F)** Decreased mRNA level of YAP, CTGF and CRY-61 were observed in overexpression experiment. Bars represents for SD. All data shows a representative of three independent experiments. * represents for p<0.05, ** represents for p<0.01.

We tested the expression level of several vital proteins in the GC cells transfected with si-lincRNA-p21. Our result showed up-regulated YAP, β-catenin and NF-κB in the si-lincRNA-p21 group, which is consistent with several recent reports [22, 23], while the expression of P-ERK was somehow decreased (Figure 5A). We found up-regulated YAP and P-YAP level in the lincRNA-p21 knockdown cells (Figure 5B). However, the expression of LATS1, the main upstream activator of YAP cytoplasmic accumulation in Hippo pathway, remained almost unchanged (Figure 5B). Next qRT-PCR assays showed that the down-regulated lincRNA-p21 expression correlated with elevated levels of YAP mRNA, connective tissue growth factor (CTGF) and cysteine-rich angiogenic inducer 61 (CYR-61), in MGC-803 cells (Figure 5C) and MKN-45 cells (Figure 5D). CTGF and CYR-61 are two targets of YAP activation [24]. Moreover, we also found lower levels of YAP and P-YAP expression in the MGC-803 cells transfected with pcDNA 3.1-lincRNA-p21 (Figure 5E). Furthermore, qRT-PCR assays also showed decreased mRNA levels of YAP, CTGF and CYR-61 in MGC-803 cells transfected with pcDNA 3.1-lincRNA-p21 (Figure 5F).

### The regulation of lincRNA-p21 on YAP nuclear translocation

**Figure 6:**
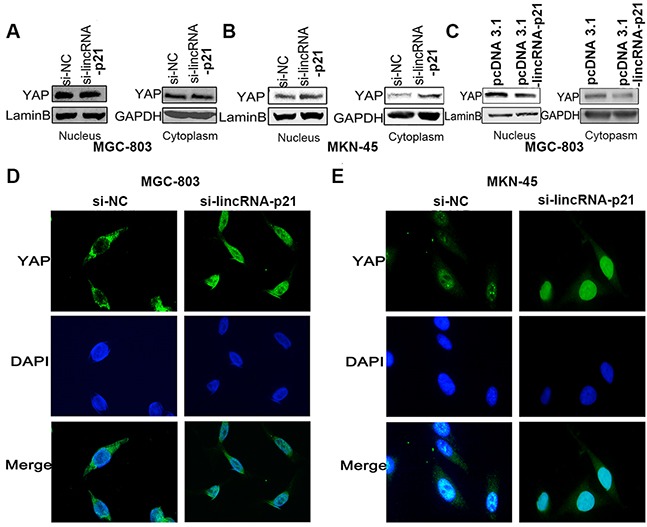
The regulation of lincRNA-p21 on YAP expression might exert through nucleus translocation The cell lysates from nucleus and cytoplasm were subjected to western-blot. In knockdown experiments, overexpression of YAP in both nucleus and cytoplasm were found in MGC-803 **(A)** and MKN-45 cells **(B)**. In overexpression experiment, down-regulated YAP expression was observed in nucleus and cytoplasm in MGC-803 cells **(C)**. IF staining demonstrated an obvious nucleus accumulation of YAP in cells transfected with si-lincRNA-p21 in MGC-803 **(D)** and MKN-45 **(E)** cells. Pictures were captured under the 40 magnification.

Besides the elevated mRNA of YAP, we wondered whether the elevated YAP protein level was correlated with increased YAP nuclear translocation. We first extracted nuclear and cytoplasmic proteins separately and analyzed them by western blot. We found increased YAP protein levels in both the nucleus and cytoplasm in two GC cell lines transfected with si-lincRNA-p21 (Figure 6A and 6B). Overexpression of linRNA-p21 showed an opposite result (Figure 6C). We also performed immunofluorescence staining experiments to visualize YAP intracellular localization. Staining revealed an obvious accumulation of YAP in the nucleus in si-lincRNA-p21 treated-cells compared to control siRNA-treated cells, in which YAP was mainly cytoplasmic (Figure 6D and 6E). We conclude that lincRNA-p21 may regulate YAP nuclear translocation.

### The involvement of lincRNA-p21 in the GC development may relate to the YAP expression

**Figure 7:**
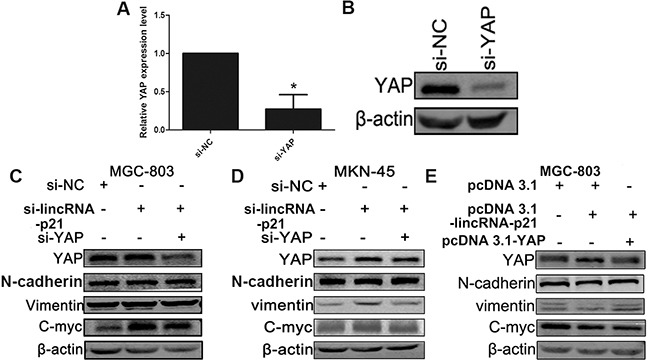
YAP played an important role in the relationship between the lincRNA-p21 and EMT process The qRT-PCR result **(A)** and the western-blot result **(B)** demonstrated the knockdown efficiency of si-YAP. MGC-803 **(C)** and MKN-45 **(D)** cells were transfected with si-NC, si-lincRNA-p21 or si-lincRNA-p21/si-YAP. Si-lincRNA-p21/si-YAP group counteracted with the overexpression of YAP induced by the knockdown of lincRNA-p21. The overexpression of N-cadherin, Vimentin and C-myc in si-lincRNA-p21 got reversed. The overexpression experiment showed an opposite result **(E)**. * represents for p<0.05

Since YAP plays an important role in tumorigenesis and triggering the EMT process [16, 17], we wondered whether the involvement of lincRNA-p21 in the GC development was related to the YAP expression. The knockdown efficiency of si-YAP was tested by both qRT-PCR (Figure 7A) and western blot (Figure 7B). Western blots indicated that cells transfected with siRNAs targeting both lincRNA-p21 and YAP counteracted the induction of YAP, N-cadherin, Vimentin and C-myc observed in single si-lincRNA-p21 transfection group in both MGC-803 (Figure 7C) and MKN-45 (Figure 7D) cells. Overexpression experiments validated these observations (Figure 7E). In this case, we came to the conclusion that the down-regulation of lincRNA-p21 contributes to GC development, possibly through the upregulation of YAP.

## DISCUSSION

The high incidence rate and relative low survival rate makes gastric cancer a significant threat to human health. However, GC is a multistep carcinogenesis process which involves genetic alterations, epigenetic changes and multiple oncogenic pathways. Thus understanding the mechanism of GC development is urgent for us to discover new potential targets for effective therapy.

LncRNAs have become focal points in studying cancer development and progression due to their extensive regulation in cells and roles as signals, decoys, guides and scaffolds [25, 26]. LincRNA-p21 was first described by the *Huarte* lab as a tumor suppressive noncoding RNA with a length of ∼3kb. It could regulate the cell cycle and apoptosis as a direct transcriptional target of p53 [8]. Since abnormal p53 gene status contributes to the progression of various tumors, the involvement of lincRNA-p21 in carcinogenesis has attracted much attention. During the analysis of the expression pattern of lincRNA-p21 in GC tissues, we found a significantly decreased lincRNA-p21 level in the gastric tissues compared with normal tissues. What's more, the clinicopathological feature analysis revealed that the lower level of lincRNA-p21 was correlated with the higher invasion depth grade, more distant metastasis incidence and more advanced TNM stage, implying lincRNA-p21′s role as a potential prognostic biomarker and a therapeutic target for gastric cancer. Meanwhile, we also compared the lincRNA-p21 expression between the GC cell lines and normal gastric mucosal cell line (GES-1). We found decreased lincRNA-p21 expression in GC cell lines, which is consistent with the lower expression levels observed in tumor tissues. To our knowledge, this is the first report of the correlation between lincRNA-p21 expression and gastric cancer progression.

The lncRNAs have been proved to engage in cancer metastasis such as HOTAIR, MALAT1 and HULC [27–29]. Our *in vitro* experiments showed morphology alteration and up-regulated of N-cadherin and Vimentin expression in si-lincRNA-p21-treated cells. We also found that knocking down lincRNA-p21 expression promoted cell migration and invasion of GC cell lines. Overexpression of linRNA-p21 verified the correlation between lincRNA-p21 and the EMT process.

YAP, the key component of the Hippo pathway, has been well studied for its implication in organ size control and stem cell differentiation [19, 20]. The Hippo pathway was originally identified in *Drosophila* and is comprised of a series of phosphorylation cascades [30–32]. LATS-mediated phosphorylation of YAP down-regulates its expression by promoting its cytoplasmic accumulation and the ubiquitination as well as the subsequent proteasomal degradation [33]. However, the expression of YAP is not merely regulated by Hippo-dependent manner [34–36]. More importantly, the finding of the inter-link between YAP and microRNA biogenesis revealed a potential regulatory role of noncoding RNA in YAP expression [37]. We demonstrated that knockdown of the lincRNA-p21 induced the elevation of both protein and mRNA levels of YAP in a Hippo-independent manner. Furthermore, overexpressing lincRNA-p21 reversed this effect. Intriguingly, we also observed increased YAP expression in both the cytoplasm and nucleus in si-lincRNA-p21 treatments cells. However, the underlying molecular mechanism of the YAP expression alteration induced by changes in lincRNA-p21 expression still needs further elucidation.

In conclusion, the lincRNA-p21 could be regarded as a tumor suppressor in gastric cancer due to its significantly decreased expression in cancer tissues. In addition, the relatively low level of lincRNA-p21 is correlated with higher invasion depth and more distant metastasis incidence rate as well as more advanced TNM stage. Moreover, lincRNA-p21 could impair cancer cell proliferation, migration and invasion ability. This signature is related to the alteration of YAP expression. Most importantly, this elevation is the result of both the up-regulated mRNA level and increased nuclear translocation of YAP.

## MATERIALS AND METHODS

### Specimens and relative clinical data

40 pairs of fresh GC tissues and adjacent non-tumor tissues along with the patients’ information were acquired from the tissue bank of West China Hospital, Sichuan University, China. The tissues were stored in liquid nitrogen for further RNA extraction. This study was approved by the Research Ethics Committee of West China Hospital. The patients’ baseline information was listed in Table 1.

### Cell lines

Five gastric cancer cell lines (MGC-803, MKN-45, BGC-823, MKN-28, SGC-7901) and a normal gastric mucosal cell line (GES-1) were obtained from the cell depository of our laboratory. These cell lines were cultured in Dulbecco's modified Eagle (DMEM) (GIBCO, USA) supplemented with 10% fetal bovine serum (FBS) (GIBCO, USA) in a humid atmosphere of 5% CO2 at 37%.

### RNA extraction and quantitative real time-PCR analysis

The total RNA of patients’ tissue specimen and cells were both extracted using the NucleoZOL reagent (MACHEREY-NAGEL, Germany) in accordance to the protocol. The concentration and quality of RNA was detected by NanoDrop. The extracted RNA was converted to cDNA by using PrimeScript™ RT reagent Kit with gDNA Eraser (Takara, Japan) following the manufacturer's instruction. The cDNA template was mixed with gene specific primers as well as SYBR Green 2X mixture (Bio-Rad, CA, USA). Quantitative real time-polymerase chain reaction (qRT-PCR) was carried out on Bio-Rad CFX96 Touch (Bio-Rad, USA). The primer sequences were listed in Supplementary Table 1. The expression level of target mRNA was calculated by 2^−ΔΔCT^ values normalized to GAPDH.

### RNA interference and plasmid transfection

The small interfering (si) RNA targeted linc-RNA-p21 was designed as same as the known sequence in a former report [38]. Both the si-lincRNA-p21 and a non-specific si-NC as a negative control were both purchased from GenePharma (GenePharma, China). The sequence of the siRNA was listed in Supplementary Table 1. Plasmid used for overexpression, pcDNA 3.1-lincRNA-p21, was constructed and sequencing confirmed by Sangon Company (Sangong, China). MGC-803 and MKN-45 were seeded in a 6-well plate 24h before transfection at approximately 1×10^5^ cells/per well. Then either si-lincRNA-p21 or si-NC was transfected using Lipofectamine 2000 (Invitrogen, USA) under the guidance of the protocol. The newly constructed plasmid was extracted by EndoFree Plasmid Mini Kit (CWBIO, China) follow the instruction. We used pcDNA 3.1 as the internal control which was directly obtained from the stock of the lab. 48h later, the transfected cells were harvested for further analysis.

### Cell counting Kit-8 assay

Cell proliferation was tested by Cell Counting Kit-8 (CCK-8) (Dojindo, China) following the manufacturer's instructions. MGC-803 and MKN-45 were seeded in a 96-well plate 24h before the transfection of si-lincRNA-p21 or si-NC. 100μl of serum free DMEM contains 10% CCK-8 reagent was added into each well at 0h and 48h post transfection respectively and then cultured for 1h. The absorbance was assessed at a wavelength of 450nm by a microplate reader (Bio-Rad, USA). Experiments were repeated at least three times.

### Ethynyl-2-deoxyuridine (EdU) incorporation assay

Cell proliferation ability was also tested by EdU Apollo DNA *in vitro* kit (RIBOBIO, China) according to instructions. MGC-803 and MKN-45 were seeded in a 96-well plate 24h before the transfection of either si-lincRNA-p21 or si-NC. At the 48h time point, 100 μl of 50 μM EdU was added in each well and incubated for 2 h at 37%. After that, the cells were fixed in 100ul of 4% paraformaldehyde (prepared in PBS) for 30 minutes at room temperature. The cells were incubated in 50 μl of 2 mg/ml glycine for 5 minutes followed by PBS washing. Afterwards, the cells were permeabilized with 1% TritonX and reacted with 1X Apollo solution for 30 minutes at the room temperature in dark subsequently. Finally, 40μl of Hoechst solution was added in each well and incubated for 15 minutes at room temperature in dark with PBS washing followed. In the end, the cells were visualized and photographed under a fluorescence microscopy. Experiments were repeated at least three times.

### Transwell and migration assay

MGC-803 and MKN-45 were seeded in a 6-well plate 24h before the transfection of either si-lincRNA-p21 or si-NC. At the 48h time point, the transfected cells were harvested and starved in serum free DMEM and then placed in the upper chambers with or without matrigel coated in duplicate. The lower chambers were filled with DMEM containing 20% FBS. After wiping off the cells on the upper surface by wet cotton swab, the cells migrated to the lower surface of the chamber were fixed in 4% paraformaldehyde and stained with 0.5% crystal violet solution and then photographed under the microscope. Experiments were repeated at least three times.

### Wound healing assay

MGC-803 and MKN-45 were seeded in a 6-well plate 24h before the transfection of either si-lincRNA-p21 or si-NC. Right after the transfection, a scratch was made through the center of each well using the 200ul sterile pipette tip. The scratch was observed and pictured at 0h, 24h, 48h post-transfection. Experiments were repeated at least three times.

### Western blot assay

Cellular protein extracts were obtained from cultured cells with RIPA lysis buffer (Bioteke, Beijing, China). The Nuclear and Cytoplasmic Extraction Kit (KeyGEN, Jiangsu, China) was used to prepare cytoplasmic and nuclear extracts separately according to the manufacture's instruction. The protein extracts were separated by 8% SDS-polyacrylamide gels and transferred electrophoretically onto a PVDF membrane using the Bio-Rad semidry transfer system. Membranes were blocked with 5% milk overnight and incubated with primary antibodies (diluted in 1:1000) for 2 hours at room temperature. Second antibodies (diluted in 1:100) were added after washing and incubated for 1 hour in dark. After washing again, the membranes were exposed in a dark room using machine. All of the primary antibodies and secondary antibodies were purchased from the Abcam campany.

### Fluorescence microscopy

MGC-803 and MKN-45 cells were transfected by either si-lincRNA-p21 or si-NC 48hrs before plating into 24-well dishes containing 12 mm glass coverslips. After 24hrs, cells were fixed in 4% paraformaldehyde and then incubated 1 h in blocking buffer (0.5% triton X-100 and 1% BSA in PBS) prior to primary antibody staining. Both primary and secondary antibodies were diluted in blocking buffer. Coverslips were incubated with primary antibodies for 1 h at room temperature followed by 1h second antibodies incubation. In the end, diluted DAPI was added on the coverslips. 10 minutes later, coverslips were mounted on the glass slide. Images were captured under Nikon Eclipse 80i (Nikon, Japan) microscope with NIS-Elements software (version 4.30.01).

### Statistical analysis

The analysis between clinicopathological characteristics and lincRNA-p21 expression was performed using SPSS 22.0 software (IBM, USA). The rest data analysis was performed using Graphpad Prism 5.0. Student t test was used for the comparison between two groups and Chi-square test or Fisher's exact test for analyzing the correlation between lincRNA-p21 expression level and patient's clinicopathological characteristics. Quantitative analysis from pictures was performed by ImageJ v1.04 software (NIH, Bethesda, MD). All of the *p* values were two-sided and *p* values less than 0.05 were considered to be statistically significant.

## SUPPLEMENTARY MATERIALS TABLES


