# Supplementary material for: Down regulation of lincRNA-p21 contributes to gastric cancer development through Hippo-independent activation of YAP
Source: Oncotarget. 2017 Jul 10;8(38):63813–24. doi: 10.18632/oncotarget.19130 (PMC5609963; doi:10.18632/oncotarget.19130)
Supplement: Supplementary file 1 [file oncotarget-08-63813-s001.pdf]

Down regulation of lincRNA-p21 contributes to gastric cancer development through Hippo-independent activation of YAP

SUPPLEMENTARY TABLE

Supplementary Table 1: Primer sequences for qRT-PCR

Name	Sequence(5'-3')
si-lincRNA p21	GCAUUGUUGCAUCAUCAUG
lincRNA-p21 F	GGGTGGCTCACTCTTCTGGC
lincRNA-p21 R	TGGCCTTGCCCGGGCTTGTC
GAPDH F	CAAGGCCAACCGCGAGAA
GAPDH R	CCCTCGTAGATGGGCACAGT
YAP F	GGTGCCACTGTTAAGGAAAGG
YAP R	GTGAGGCCACAGGAGTTAGC
CTGF F	TGGTGCAGCCAGAAAGCTC
CTGF R	CCAATGACAACGCCTCCTG
Cyr-61 F	TTCTTTCACAAGGCGGCACTC
Cyr-61 R	AGCCTCGCATCCTATACAACC
